# Supplementary material for: Gradient descent in materia through homodyne gradient extraction
Source: Nat Commun. 2025 Nov 21;16:10272. doi: 10.1038/s41467-025-65155-7 (PMC12639101; doi:10.1038/s41467-025-65155-7)
Supplement: Supplementary file 1 — Supplementary Information [file 41467_2025_65155_MOESM1_ESM.pdf]

Supplementary Information for

Gradient descent *in materia*

through homodyne gradient extraction

Marcus N. Boon[1,2,3*], Lorenzo Cassola[1,4*], Hans-Christian Ruiz Euler[1], Tao Chen[1], Bram van de Ven[1], Unai Alegre Ibarra[1], Peter A. Bobbert[1,5], Wilfred G. van der Wiel[1,4†]

[1]NanoElectronics Group, MESA+ Institute for Nanotechnology and BRAINS Center for Brain-Inspired Computing, University of Twente, PO Box 217, Enschede 7500 AE, The Netherlands.

[2]Exzellenzcluster Science of Intelligence, Technische Universität Berlin, Marchstr. 23, 10587 Berlin, Germany.

[3]Department for Electrical Engineering and Computer Science, Modeling of Cognitive Processes, Technische Universität Berlin, Berlin, Germany.

[4]Institute of Physics, University of Münster, 48149 Münster, Germany.

[5]Molecular Materials and Nanosystems & Eindhoven Institute for Renewable Energy Systems, Department of Applied Physics, Eindhoven University of Technology, PO Box 513, Eindhoven 5600 MB, The Netherlands.

*These authors contributed equally.

[†]Correspondence to: W.G.vanderWiel@utwente.nl

Table of Contents

**Supplementary Note 1**	Gradient estimation for large perturbation amplitudes
**Supplementary Note 2**	RNPU output response ratios for perturbed inputs with neighbouring frequencies
**Supplementary Note 3**	Alternative training methods for RNPUs
**Supplementary Note 4**	Comparison of HGE with simultaneous perturbation stochastic approximation (SPSA)
**Supplementary Note 5**	Backpropagating through a multi-RNPU system

Supplementary Note 1. Gradient estimation for large perturbation amplitudes

Increasing the perturbation amplitude has a clear benefit for the accuracy of the estimated derivative: it increases the output signal-to-noise ratio. However, increasing the amplitude too much may violate the linearity assumption of the first-order Taylor expansion (see Methods). When this occurs, the gradient estimation becomes biased, offsetting the gradient descent direction for both finite differences (FD) and homodyne gradient extraction (HGE). Thus, increasing the perturbation amplitude is often unsuitable for improving derivative accuracy. An example of this bias is shown in Supplementary Fig. 1 for a nonlinear test function $h(w)$.

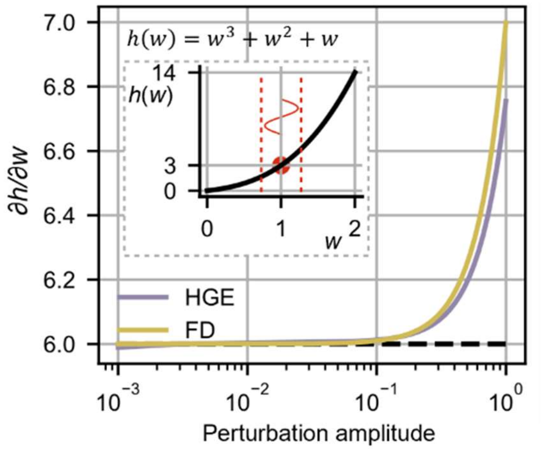

Supplementary Figure 1. Simulated derivatives. Simulated finite differences (FD) and homodyne gradient extraction (HGE) derivatives for increasing perturbation amplitude in the case of a nonlinear test function $h(w) = w^3 + w^2 + w$, w = 1 + perturbation), introducing an increasing bias to the derivative. The inset illustrates the point of the function where HGE and FD are applied and the perturbation amplitude.

Supplementary Note 2. RNPU output response ratios for perturbed inputs with neighbouring frequencies

In the main text we discuss what the frequency spacing between the distinct perturbations should be to perform parallel HGE in dopant network processing units (RNPUs) without loss of accuracy. In the Methods section, we describe an analytical procedure for estimating the minimal required frequency spacing, based on RNPU-specific parameters, such as the noise magnitude and the parameters of the low-pass filter. In addition to those parameters, information about the typical expected output response to the input perturbations (see Methods) is also necessary to obtain an accurate estimate of the minimal required frequency spacing. In this section, we describe how we obtain this information.

To obtain an estimate of the output responses for all input electrodes, we apply 1,000 randomly chosen input voltage combinations to the electrodes, with voltages in the range [−1 V, 1 V], and perturbations using a frequency spacing of 40 Hz and a highest frequency of 1 kHz. The perturbation amplitudes for the inputs of the device shown in Fig. 2a in the main text are [20, 30, 30, 30, 30, 30, 20] mV (smaller perturbations of 20 mV are applied to the electrodes 1 and 7 closest to the output, which have a larger influence on the output current). By performing a Fourier transform of the output current, we isolate the current amplitudes I_n (see Eq. (5) in Methods) of the response in the output current to the perturbation of electrode n. To quantify the expected error coming from parallel perturbations, we determine for each input the ratio of the amplitude I_n of the output current and the amplitude $I_{n'}$ of input n' of an electrode with a directly neighbouring perturbation frequency (we take the inverse ratio in case this ratio is smaller than 1). To determine a realistic scenario and a worst-case scenario, we determine the median value of all ratios and the ratio that delimits the 1st percentile of largest ratios, respectively. The median ratio is 2.05 and the 1st percentile ratio is 19.57. Supplementary Figure 2 shows the histogram of all ratios.

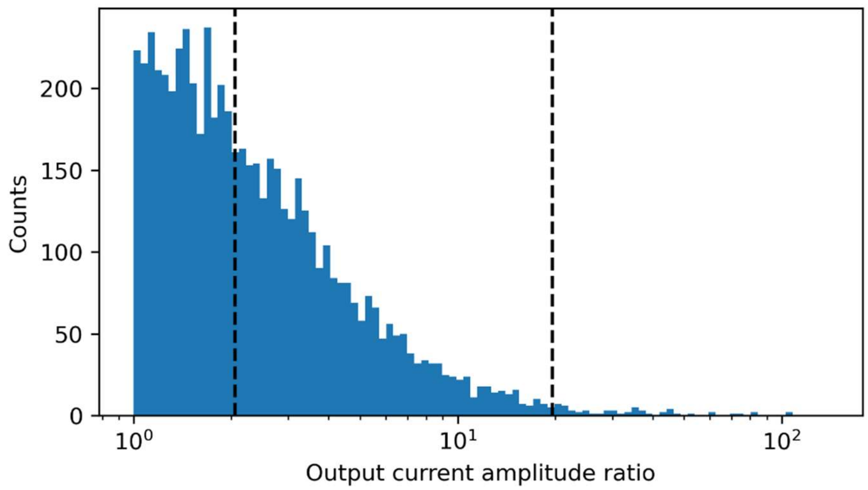

Supplementary Figure 2. Statistics for the output current amplitude. Histogram of the ratios of the output current amplitudes of inputs perturbed with neighbouring frequencies (40 Hz apart), determined for 1,000 combinations of input voltages. The inverse ratio is taken in case the ratio is smaller than 1. The vertical dashed lines indicate the median ratio of 2.05 and the first percentile ratio of 19.57, respectively.

Supplementary Note 3. Alternative training methods for RNPUs

As stated in the main text, we used different approaches in the past to train RNPUs, such as a genetic algorithm (GA) [20] and in-silico training [12]. Here we compare these approaches to HGE, demonstrating the practical advantages of HGE for RNPU optimization. For a detailed description of a GA applied to RNPUs we refer to Ref. [20]. While a GA arguably performs a broader search in the space of all possible solutions, it generally tends to converge worse to local minima than gradient-based approaches, potentially leading to less accurate results. Furthermore, depending on the objective landscape, it can come with increased computational costs. To demonstrate this in our devices, we applied a GA in finding solutions for both the XNOR Boolean logic gate and a 2D ring classifier task, described in Ref. [12], in the device shown in Fig. 2a of the main text. We chose these two tasks as benchmarks since for the former we are confident that a solution can be found in the RNPU (as previously shown in Refs. [12] and [20]) without being trivial (it is the most complicated Boolean gate for the device taken into consideration, measured by the number

of generations to obtain a given fitness), while the latter task is more challenging and requires precise tuning to obtain accurate results.

We solved the XNOR Boolean logic task 10 times (100 % accuracy was reached for every run) with both a GA (20 genomes) and HGE. In Supplementary Table 1 we report the total time and number of iterations needed to obtain 100% accuracy for these 10 independent runs.

Supplementary Table 1. GA and HGE convergence times and number of iterations needed for solving the XNOR gate on a RNPU. Data for 10 independent runs on the RNPU presented in Fig. 2a, using 20 genomes for the GA.

Run #	Time GA (s) (nr. iterations)	Time HGE (s) (nr. iterations)
1	209.2 (26)	56.9 (97)
2	81.3 (10)	12.5 (21)
3	113.6 (14)	17.6 (30)
4	66.3 (8)	147.8 (257)
5	433.8 (54)	11.3 (19)
6	146.6 (18)	46.8 (81)
7	129.9 (16)	97.9 (170)
8	130.0 (16)	61.2 (106)
9	257.8 (32)	31.9 (55)
10	154.9 (19)	4.4 (7)

While the task can be solved with both methods, HGE is considerably faster than the GA (Supplementary Table 1). Specifically, when comparing both the average (172.3 s for the GA and 48.8 s for HGE) and the median (138.3 s for the GA and 39.4 s for HGE) training times, HGE accelerates the process by more than three times compared to the GA. Next, we applied a GA and HGE to the 2D ring classification task of Ref. [18], which can be considered as a simplified version of the 3D sphere classification task, which was solved by HGE as reported in the main text. We were not able to find any good solution when using a GA, starting 60 times from a different random combination of control voltages, whereas with gradient descent in a

surrogate model good solutions were readily found [12]. We expect that this discrepancy is due to the sparseness of the solution in parameter pace. As mentioned above, GA is less suitable for tasks where the solution requires precise localization of a local minimum. The two comparisons we carried out on RNPUs show how HGE outperforms GA, both in terms of speed and of complexity of the classification tasks that can be solved.

In-silico training (using a surrogate neural network model) is the only other method that has been shown to be effective for training RNPUs for complex classification tasks [12]. However, a direct comparison with on-chip methods such as HGE and GA is nontrivial as it is impossible to establish fair comparative conditions. Since in-silico training first requires the acquisition of data and training of a surrogate model, there is a large overhead before the optimization procedure can start. This data acquisition takes many hours, the exact time depending on the scanning speed of the parameter space. If this procedure needs to be performed merely to search for a single solution (as is done in this section), it is clear that in-silico training is orders of magnitude slower than on-chip methods. If, however, it is assumed that this model can afterwards be used indefinitely (i.e., the device does not change its IV-characteristics or breaks down entirely) and many tasks are required to be solved, a surrogate model might provide an advantage. Nonetheless, in the latter case the surrogate model can at best provide solutions with similar accuracies as HGE, provided that the model precisely matches the real device. Furthermore, for in-silico training an external system would always be required, which is more energy intensive than on-chip training and therefore ultimately undesirable.

Supplementary Note 4. Comparison of HGE with simultaneous perturbation stochastic approximation (SPSA)

To evaluate the performance of HGE relative to established zeroth-order optimization baselines, we conducted a series of controlled simulations comparing HGE to simultaneous perturbation stochastic approximation (SPSA). We decided to compare HGE to SPSA here instead of finite difference (FD) since the latter is considered impractical in realistic optimization problems. We included three variants of SPSA:

- 2-sample SPSA (the standard approach), in which we use two samples to estimate the gradient.
- 10-sample SPSA, in which we estimate the SPSA gradient 5 times and take the average gradient.
- An adaptive *N*-sample SPSA, where *N* matches the number of samples used in HGE.

We decided to include these three variants of SPSA since the first is the basic version of SPSA, the second utilizes averaging of gradient estimations, which is often used when the function is noisy, and the third additionally directly matches the per-iteration computational budget of our proposed HGE method (the value of *N* is explained below). In our comparison, we addressed both the gradient quality of a single gradient estimation and the full optimization performance under various noise conditions and for increasing numbers of input parameters. We used a network of coupled nonlinear oscillators as a benchmark system:

$$F(x) = \sum_{i=1}^{p} \frac{(x_i - x_{i-1})^2}{2} + \frac{(x_i - x_{i-1})^4}{4}, \text{with periodic boundary } x_0 = x_p,$$

where p is the number of input parameters. The network of coupled nonlinear oscillators provides a practical benchmark task for optimization since it is inherently nonlinear and is easily scalable to higher dimensions.

To optimally utilize the properties of HGE, we decided to use each perturbation frequency for two different input parameters, where one parameter is perturbed with a zero-phase waveform, and the other parameter is perturbed with a 90-degrees shifted waveform. By only extracting the X-component in the demodulation step of lock-in detection (see Methods), we effectively utilize phase information to increase the number of waveforms that we can fit within the frequency band.

The number of samples for N-sample SPSA and HGE depends both on the dimensionality of the benchmark task and the magnitude of the noise. For both factors, a minimum required number of samples is determined by some simple derivations, and the highest value of the two estimates is used for our simulations. The dependency of number of samples with respect to task dimensionality is determined as follows. The distance between the frequencies used for HGE is defined as

$$\Delta f = \frac{f_{\max} - f_{\min}}{p/2},$$

Where $f_{\max}$ and $f_{\min}$ are the highest and lowest perturbation frequencies used, and p is the number of optimizable parameters. The factor 2 originates from using each frequency twice, as explained above. To reduce interference between neighboring perturbation frequencies, we want their distance to be a multitude m of the cut-off frequency, which is picked as

$$f_c = \frac{\tau}{2\pi N},$$

where τ is a constant which is chosen such that the is filter 98% settled at the end of sampled signal. Combining the above two equations, the required number of samples to achieve the constraint $\Delta f \geq m \times f_c$ is given by

$$N = \frac{m\tau p}{4\pi(f_{\max} - f_{\min})}.$$

For a task dimensionality of p = [5, 10, 20, 50, 100, 200, 500], we use $m = 2$, $f_{\min}$ = [0.3, 0.3, 0.2, 0.1, 0.1, 0.1, 0.1], and $f_{\max} = 0.5$ (Nyquist frequency), resulting in a total number of samples per iteration of [24, 48, 64, 120, 238, 476, 1190] for a task dimensionality of p = [5, 10, 20, 50, 100, 200, 500].

The dependency of number of samples with respect to noise strength is determined as follows. Assuming Gaussian white noise, the power of the noise after filtering the output signal can be found by

using the equivalent noise bandwidth f_{enwb} (see Methods). Using f_{enwb}, the relationship between the filtered noise power and the number of samples can be described as

$$P_{\mathrm{noise}} = S_{\mathrm{n}}(f) \times f_{\mathrm{enbw}} \equiv S_{\mathrm{n}}(f) \times a \times f_{\mathrm{c}} \,,$$

$$N = \frac{a\tau S_{\mathrm{n}}(f)}{2\pi P_{\mathrm{noise}}},$$

where $S_{\mathrm{n}}(f)$ is the noise power per Hz, f_{c} is the cut-off frequency as defined above, and a is a parameter that relates f_{c} to f_{enwb} for a given filter type and order ($a \approx 1.1107$ for a second-order Butterworth filter). Given that we set $P_{\mathrm{noise}} = 0.2$, the total number of samples per iteration is [6, 12, 28, 54, 106, 266, 530] for $S_{\mathrm{n}}(f)$ = [1, 2, 5, 10, 20, 50, 100] Hz^{-1}. Note that while this analysis assumes Gaussian white noise, it can still be used to estimate an approximate number of samples for the $1/f$ noise case. Lastly, we defined a lower limit of number of samples used for a single iteration, which was set to 30.

To test the accuracy of the single gradient estimations, we uniformly sampled 100 random inputs in the range [-2, 2] in each dimension. The simulation provides access to ground-truth gradients for controlled evaluation and allows to systematically vary the dimensionality of the input space and the total power of additive $1/f$ noise on the output. The resulting relative errors, defined as $||g(x) - \hat{g}(x)||/||g(x)||$, where $g(x)$ and $\hat{g}(x)$ are the true and estimated gradient, of the three different methods used are found in Supplementary Figure 3.

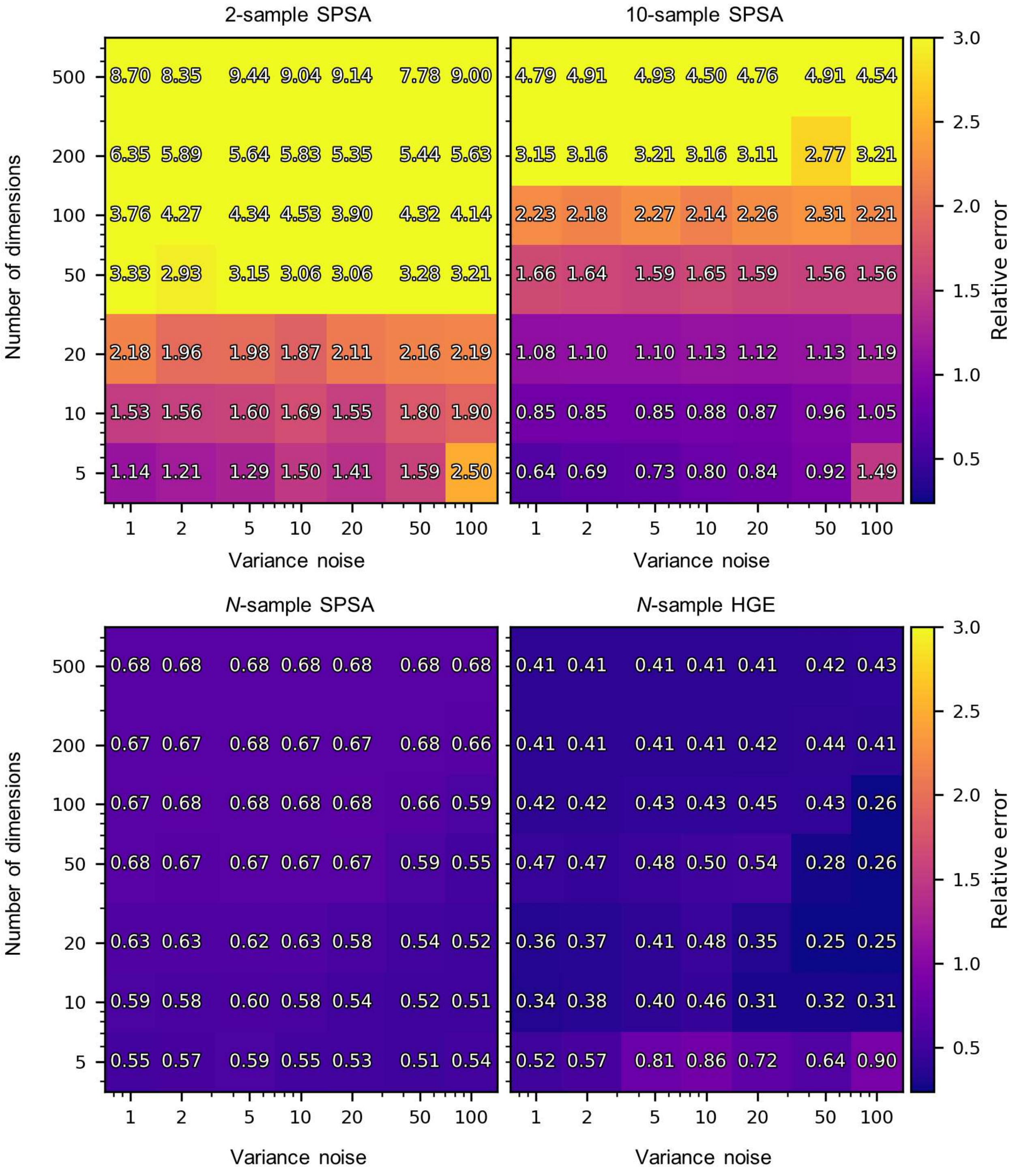

Supplementary Figure 3. Average relative error for gradient estimation using SPSA and HGE. Four different sampling and/or gradient estimation strategies are compared for each possible combination of dimensionality [5, 10, 20, 50, 100, 200, 500] and total power of $1/f$ noise signal (noise signal generated with unit variance, scaled with [1, 2, 5, 10, 20, 50, 100]). Each noise-dimension combination is run 100 times, each time randomly selecting an input vector in the range [-2, 2]. Note that the maximum value of the color bar was clipped at 3 to retain some visual overview over intermediate relative errors.

Both 2- and 10-sample SPSA have a high relative error, (often) exceeding a value of 1, indicating that the gradient is very uninformative. The adaptive *N*-sample SPSA and HGE gradients, in contrast, have much better accuracy. This is not surprising since the computational cost for estimating a single gradient is much higher than for 2- or 10-sample SPSA, and also increases with dimensionality and noise power. When comparing the *N*-sample SPSA and HGE directly, we observe that HGE has a higher accuracy for almost all settings. However, to make a fair judgement on the practicality of the methods, we instead refer to benchmark results given below, which tests how many overall samples were required for optimization.

For the full optimization procedure, we again sampled 10 random initializations in the range [-2, 2] and ran zeroth-order gradient-descent optimization for each method. Their average overall required number of samples until convergence can be found in Supplementary Figure 4. All methods were tested with identical learning rates and initial perturbation amplitudes. We used a decaying learning rate originally defined by Spall [31] as $a_k = \frac{a}{(A+k)^{\alpha}}$ with α = 0.602, a = 0.1, and A = 1, where k is the iteration number. The perturbation step size for HGE is kept constant with a value of $c = 0.1$, and the step size for SPSA is again taken from Spall: $c_k = \frac{c}{k^{\gamma}}$, where $\gamma = 0.101$. To make a fair comparison across all methods, we set a maximum overall number of samples to 500,000, regardless of task dimensionality. The stopping condition for all methods is given by $\sqrt{p}$, where p is the input dimension of the task. We explicitly decided on a dimension-dependent stopping condition since the solution space for the nonlinear coupled oscillator task very rapidly decays as the dimensionality increases. If the function evaluation exceeded a value of 1×10^{6}, we aborted the simulation and regarded the attempt as a failure due to divergence.

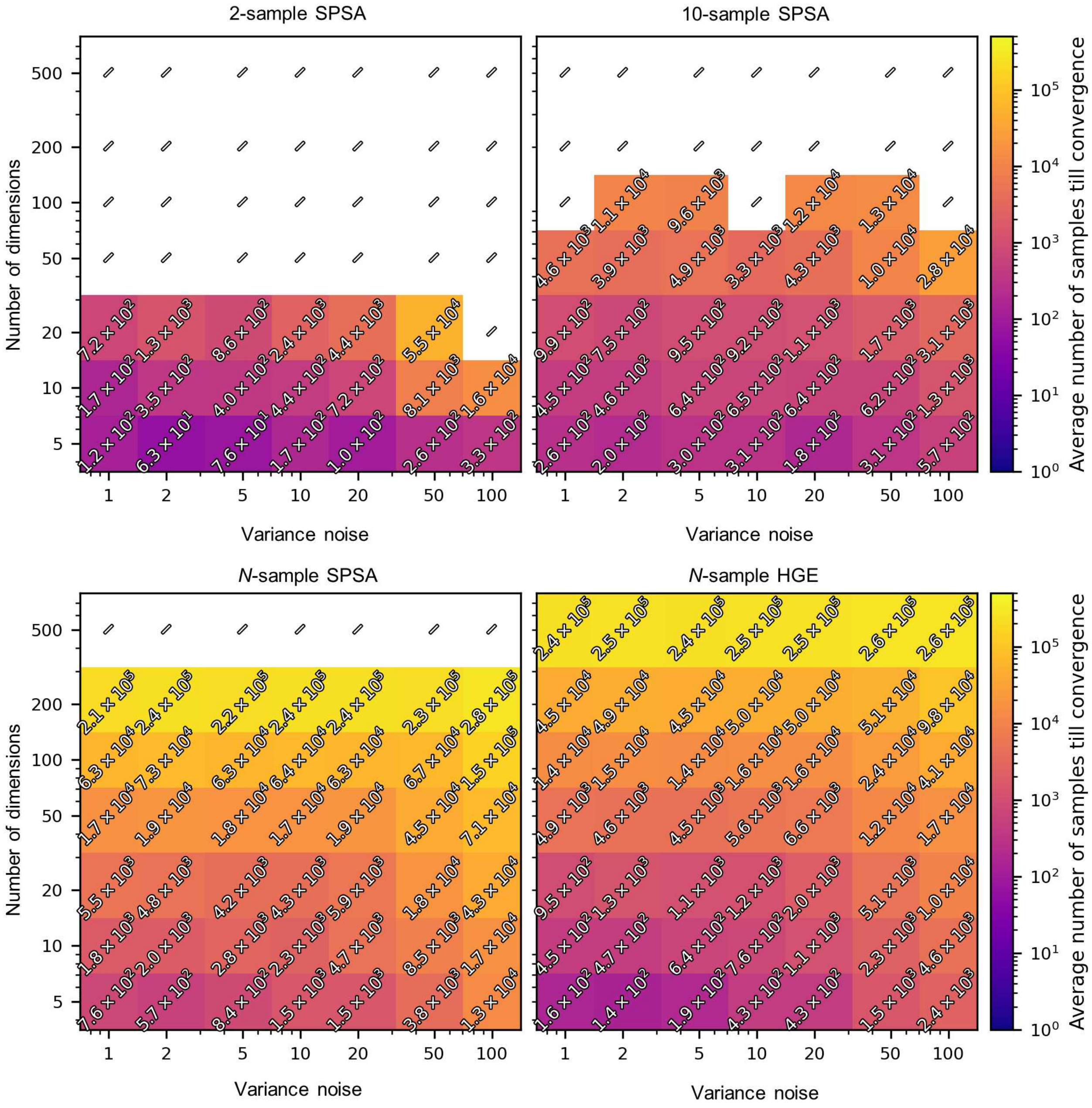

Supplementary Figure 4. Average number of samples till convergence for SPSA and HGE. Four different sampling and/or gradient estimation strategies are compared for each possible combination of dimensionality [5, 10, 20, 50, 100, 200, 500] and total power of $1/f$ noise signal (noise signal generated with unit variance, scaled with [1, 2, 5, 10, 20, 50, 100]). Each noise-dimension combination is run 10 times. If there is no value in a bin, then none of the optimization attempts converged.

We find that both 2- and 10-sample SPSA, while computationally efficient for lower dimensions, do not scale well to higher dimensions. While the adaptive N-sample HGE method is in some cases

computationally more expensive than using 2- or 10-sample SPSA, the differences are not large. Furthermore, for the adaptive method we provided two easy rules of thumb how to increase the number of samples accordingly. Using these rules we can easily scale the method to higher dimensional tasks. When comparing the *N*-sample SPSA and HGE directly with each other, we observe that HGE consistently requires a lower number of samples to converge.

Supplementary Note 5. Backpropagating through a multi-RNPU system

In the main text, we demonstrate HGE in a single RNPU device by propagating the perturbations through the entire device. For highly complex and interconnected systems, however, HGE can also be applied in a modular manner, utilizing the chain rule to multiply the gradient components similarly to backpropagation in neural networks. We illustrate this idea using RNPUs as an example.

Each RNPU can in principle be connected in a layered manner, similar to neural networks. An example of connecting two devices is shown in Supplementary Figure 5. Since the RNPUs have input voltages and their output is a current, IV-conversion is needed to interconnect the devices. Thus, the output current of the first device is measured, and a (linear) mapping between the intermediate output current and the input voltage, $V_{24}(I_1)$, is applied. At the same time, perturbations from the optimizable parameters n of the first node (which can include V_{11} up to V_{17}) are measured and their corresponding gradient parts, $\frac{\partial I_1}{\partial V_{1n}}$, are extracted using HGE. The same procedure is applied to the second node and the gradient parts $\frac{\partial I_2}{\partial V_{2n}}$ are extracted.

To now propagate the gradient back into the first node, the gradient part with respect to V_{24} is multiplied with the corresponding gradient part with respect to any of the parameters in the first node:

$$\frac{dI_2}{dV_{1n}} = \frac{\partial I_2}{\partial V_{24}} \frac{\partial V_{24}}{\partial I_1} \frac{\partial I_1}{\partial V_{1n}}, \quad (1)$$

where $\frac{\partial V_{24}}{\partial I_1}$ is a known (constant) value from the pre-defined (linear) mapping from I_1 to V_{24}. Since the partial derivatives are computed locally, we do not have the issue of losing the signal strength of the small input perturbations in a deep system, which might happen when propagating the perturbations as a single-shot method through the entire system.

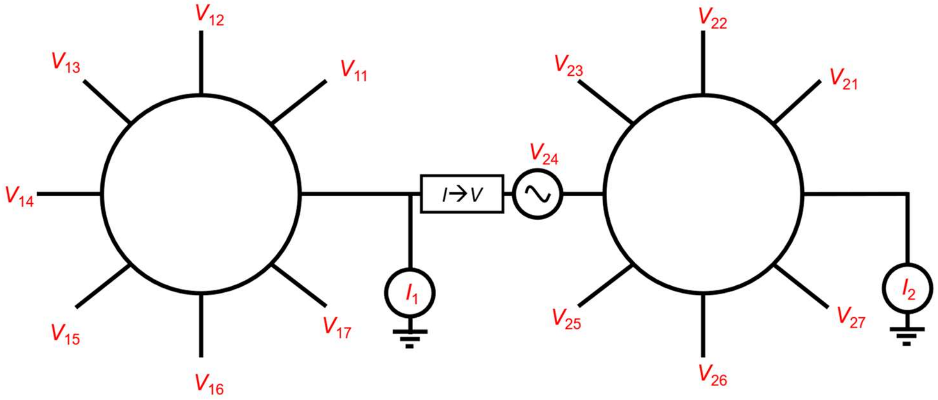

Supplementary Figure 5. Interconnected RNPUs. Schematic overview of interconnecting two reconfigurable networks. The output current of the first device is converted to a voltage in order to use its output as an input for the next device.
